# Environmental drivers of sulfur dioxide variability in a tropical urban region: a state space modeling approach for Campo Grande, Brazil

**DOI:** 10.1007/s10661-026-15377-z

**Published:** 2026-04-30

**Authors:** Amaury de Souza, José Francisco de Oliveira Júnior, Fernando Lucambio Pérez, Kelvy Rosalvo Alencar Cardoso

**Affiliations:** 1 Federal University of Mato Grosso Do Sul, C.P. 549, Campo Grande, 79070-900 MS Brazil; 2 Federal University of Alagoas. Institute of Atmospheric Sciences (ICAT), Maceió, 57072-260 Alagoas Brazil; 3 Federal University of Paraná. Block PC-09–Campus III, Polytechnic Center, Jardim das Américas, Curitiba, PR Brazil

**Keywords:** Sulfur dioxide, Air quality, State space model, Meteorology, CAMS, MERRA-2, Tropical city

## Abstract

Understanding the temporal dynamics of sulfur dioxide (SO_₂_) in tropical urban environments is essential for evaluating air quality trends and supporting environmental management policies. This study investigates daily SO_₂_ variability in Campo Grande, Brazil, from 2003 to 2022, integrating meteorological information into a multivariate autoregressive state space (MARSS) model. SO_₂_ data were obtained from SISAM/INPE (based on the CAMS global reanalysis), and meteorological covariates (precipitation, air temperature, relative humidity, and wind speed) were extracted from the MERRA-2 reanalysis. The MARSS framework was used to (i) characterize long-term and seasonal behavior, (ii) quantify the contribution of meteorological drivers to SO_₂_ variability, and (iii) identify pollution episodes not explained by atmospheric factors. SO_₂_ concentrations showed a decreasing long-term trend, with most values remaining below 10 μg m⁻^3^ and higher peaks occurring during the dry season (June–September). Including meteorological covariates improved model performance by ~ 41% (*R*^2^: 0.41–0.57). Temperature was positively associated with SO_₂_, whereas precipitation, humidity, and wind speed contributed to dilution and removal processes. Residual analyses highlighted extreme events mainly in the dry season, likely linked to atmospheric stability and episodic anthropogenic emissions such as biomass burning and local combustion sources. Overall, the results indicate that MARSS models provide a robust and interpretable approach for diagnosing SO_₂_ variability and separating meteorological control from emission-driven anomalies in tropical urban regions, supporting meteorology-informed mitigation and air quality management strategies.

## Introduction

Sulfur dioxide (SO_₂_) is a key atmospheric pollutant emitted by both natural and anthropogenic sources, including volcanic activity, biomass burning, fossil fuel combustion, industrial processes, and the oxidation of reduced sulfur compounds (Seinfeld & Pandis, 2016). In the atmosphere, SO_₂_ contributes to sulfate aerosol formation, influences cloud condensation nuclei, and affects radiative forcing, thereby playing an important role in air quality and climate interactions (Seinfeld & Pandis, 2016). From a public health perspective, short-term exposure to SO_₂_ has been associated with acute respiratory symptoms, increased hospital admissions, reduced lung function, and short-term mortality (Bascom, 1996; Fung et al., 2005; Medley et al., 2002; Park et al., 2010). These impacts support the continued relevance of monitoring and evaluating SO_₂_ variability as part of air quality management, consistent with international health-based guidelines (World Health Organization, 2021).

Recent studies based on ground-level monitoring networks indicate that sulfur dioxide (SO_₂_) concentrations in urban environments typically remain at relatively low levels, often below 10–20 μg m⁻^3^, although higher values may occur during pollution episodes associated with industrial emissions, traffic, or biomass burning. For example, Chattopadhyay et al. (2010) reported significant spatial variability of SO_₂_ in Kolkata, with elevated concentrations linked to land-use patterns and combustion sources, while Rahman et al. (2023) highlighted the importance of integrating satellite products with in situ observations to improve the representation of near-surface SO_₂_ dynamics. Similarly, studies in different urban contexts have shown that short-term peaks in SO_₂_ are strongly influenced by local emission sources and meteorological conditions, particularly during periods of atmospheric stability. Although ground-based measurements provide more accurate representations of near-surface concentrations, their spatial coverage remains limited in many regions, especially in developing countries. In this context, reanalysis products such as CAMS, which assimilate satellite retrievals and available observations, offer a valuable alternative for long-term assessments of SO_₂_ variability, particularly when the focus is on temporal behavior and meteorological modulation rather than absolute concentration values.

In Brazil, the assessment of SO_₂_ remains challenging due to the limited spatial coverage of ground-based monitoring networks, particularly outside major metropolitan regions. This limitation is critical in medium-sized tropical cities and in regions undergoing rapid land-use change, where pollutant dynamics may be strongly influenced by seasonal biomass burning and evolving combustion-related emissions. In the Center-West region of Brazil, including the state of Mato Grosso do Sul, the pronounced dry season typical of tropical savanna climates enhances pollutant accumulation through reduced wet deposition, frequent atmospheric stability, and weaker boundary-layer development. These conditions make Campo Grande a representative tropical urban area for investigating the combined influence of meteorology and emission patterns on SO_₂_ variability.

In recent years, the increasing availability of satellite-derived products and atmospheric reanalyses has provided an important opportunity to evaluate air pollution behavior in regions with scarce in situ measurements. The Copernicus Atmosphere Monitoring Service (CAMS) global reanalysis integrates satellite retrievals, ground observations, and numerical modeling within the ECMWF forecasting system, providing multidecadal datasets for reactive gases and aerosols (Christophe et al., 2019; Eskes et al., 2015; Inness et al., 2019). Likewise, meteorological reanalyses such as NASA’s MERRA-2 offer physically consistent fields of precipitation, temperature, humidity, and wind, supporting long-term environmental diagnostics and modeling (Gelaro et al., 2017). In Brazil, platforms such as SISAM/INPE provide access to CAMS-based pollutant estimates and facilitate temporal assessments of air quality indicators (Instituto Nacional de Pesquisas Espaciais, 2024).

Time-series approaches have been widely applied to air pollution studies; however, many methods rely on static assumptions that may not fully represent evolving atmospheric dynamics in tropical environments. Spatial techniques such as land-use regression (LUR), kriging, and spatial autoregressive models have been successfully used to characterize intraurban variability of pollutants such as SO_₂_, NO_₂_, and PM_₂.₅_ (Gulliver et al., 2011; Hoek et al., 2008; Kanaroglou et al., 2013; Mercer et al., 2011). Although these approaches are valuable for spatial mapping, their ability to explicitly decompose temporal processes and isolate latent dynamics is often limited when the objective is to diagnose long-term changes and seasonal controls.

In contrast, state-space models provide a flexible framework to represent environmental time series as a combination of latent components (e.g., level/trend and stochastic variability) while allowing the inclusion of exogenous predictors such as meteorological covariates (Harvey, 1989). This structure is particularly advantageous in tropical regions, where pollutant concentrations respond strongly to rainfall regimes, boundary-layer variability, and seasonal atmospheric stability. The multivariate-autoregressive state space (MARSS) formulation has gained increasing attention in environmental applications because it supports robust parameter estimation via Kalman filtering and provides interpretable diagnostics for trend detection and anomaly identification (Holmes & Scheuerell, 2022). Recent applications have demonstrated the utility of state-space methods for air pollution time-series analysis, particularly for disentangling meteorological modulation from unexplained variability (Abdul Rahim & Masseran, 2023).

Despite these advances, studies focusing specifically on long-term SO_₂_ variability in tropical South America remain limited, especially those integrating CAMS-based products with meteorological reanalysis within a state space framework. Addressing this gap is important for improving air quality diagnostics in regions where monitoring infrastructure is sparse and where seasonal biomass burning and urban expansion can intensify pollution episodes.

Therefore, this study investigates the temporal dynamics of daily SO₂ concentrations in Campo Grande, Brazil, from 2003 to 2022 using a MARSS modeling approach. The objectives are to (i) characterize long-term behavior and seasonal patterns of SO_₂_; (ii) quantify the contribution of precipitation, air temperature, relative humidity, and wind speed to daily SO_₂_ variability; and (iii) identify extreme pollution episodes not explained by meteorological conditions. By integrating environmental interpretation with state-space modeling, the study provides evidence to support meteorology-informed air quality assessment and management strategies in tropical urban environments.

## Materials and methods

### Study area

Campo Grande, the capital of Mato Grosso do Sul, Brazil (20°27′ S; 54°37′ W), is located in a tropical savanna climate zone (Aw, Köppen classification). The region is characterized by a marked seasonality, with a rainy season from October to April and a dry season from May to September. Annual precipitation averages approximately 1500 mm, and mean temperatures range between 22 and 26 °C.

These climatic characteristics strongly influence atmospheric dispersion and pollutant accumulation. During the dry season, reduced humidity, frequent thermal inversions, and weak boundary-layer development favor the buildup of combustion-related pollutants, including sulfur dioxide (SO_₂_). Campo Grande also experiences recurring biomass burning in its surroundings, rapid urban expansion, and increasing vehicle use, making it a representative tropical urban environment for assessing meteorological controls on pollutant behavior.

### Air quality and meteorological data

#### SO_2_ data

Daily SO_₂_ concentrations for 2003–2022 were obtained from the Integrated Environmental Health Information System (SISAM), maintained by the Brazilian National Institute for Space Research (INPE). SISAM SO_₂_ estimates are derived from the Copernicus Atmosphere Monitoring Service (CAMS) global reanalysis, which integrates satellite retrievals, in situ measurements, and numerical atmospheric models using the ECMWF Integrated Forecasting System.

The CAMS reanalysis provides SO_₂_ mass concentrations at 0.25° × 0.25° spatial resolution and undergoes continuous validation through international monitoring networks and aircraft campaigns (Christophe et al., 2019; Eskes et al., 2015).

### Meteorological variables

Meteorological covariates were extracted from NASA’s Modern-Era Retrospective Analysis for Research and Applications Version 2 (MERRA-2), produced by the Global Modeling and Assimilation Office (GMAO). The following daily variables were included:

- Precipitation (mm)
- Two-meter air temperature (°C)
- Relative humidity (%)
- Ten-meter wind speed (m s⁻.^1^)

These variables were selected because of their well-established physical influence on SO_₂_ dynamics through dilution, transport, wet deposition, and atmospheric stability. MERRA-2 provides the same spatial resolution as CAMS, ensuring consistency between datasets.

All time series were complete and contained no missing values. Prior to modeling, variables were standardized using *z*-scores to reduce scale differences and improve parameter estimation.

### MARSS modeling framework

A multivariate autoregressive state space (MARSS) model was used to characterize the temporal dynamics of daily SO_₂_ concentrations and to quantify the influence of meteorological drivers. State space models are particularly suitable for air quality time series because they explicitly separate the observed signal into latent components (e.g., slowly varying level/trend) and stochastic variability, while allowing the inclusion of exogenous predictors.

The MARSS model is represented by two stochastic equations: a state equation describing the evolution of the latent process and an observation equation linking the latent state to the measured SO_₂_ concentrations. In this study, SO_₂_ was treated as a univariate response series, and meteorological variables were included as external covariates to explain short-term variability.

Two model configurations were fitted and compared:

(i) Baseline model (meteorology-free): SO_₂_ dynamics were modeled without covariates, capturing the latent level/trend and stochastic components only.
(ii) Meteorology-driven model (extended): precipitation, air temperature, relative humidity, and wind speed were included as covariates to quantify their contribution to SO_₂_ variability.

All covariates were standardized (*z*-scores) prior to model fitting to ensure comparability of regression coefficients and to improve numerical stability during estimation. Parameters were estimated by maximum likelihood using the Kalman filter and Kalman smoother, as implemented in the MARSS package for R (Holmes & Scheuerell, 2022). Model outputs were interpreted in terms of long-term evolution (latent level), seasonal behavior, and the sign and magnitude of meteorological effects.

### Model evaluation and diagnostics

Model performance was evaluated by comparing the baseline and extended MARSS configurations using the coefficient of determination (*R*^2^), computed from observed and model-predicted SO_₂_ concentrations. The improvement obtained by including meteorological covariates was quantified as the relative gain in explained variance.

Residual diagnostics were conducted to assess whether the fitted models adequately captured the temporal structure of the SO_₂_ series. Temporal autocorrelation in residuals was examined using the Ljung–Box test (lag 30). The distributional behavior of residuals was evaluated using the Kolmogorov–Smirnov and Anderson–Darling tests, complemented by visual inspection of standardized residuals.

To identify extreme SO_₂_ pollution episodes not explained by meteorological variability, residuals from the extended model were standardized and screened for values exceeding the 99th percentile. These exceedances were interpreted as anomalous events potentially associated with episodic emission sources (e.g., biomass burning or local combustion activities) or atmospheric conditions not fully represented by the selected covariates.

### Cross-validation

To assess predictive robustness, a time-ordered cross-validation procedure was performed. The dataset was split chronologically into two subsets: the first 80% of observations were used for model calibration (training), and the remaining 20% were reserved for validation (testing). This temporal partitioning preserves the sequential structure of the series and avoids using future information to estimate past states.

Model performance in the validation period was evaluated by comparing observed and predicted SO₂ concentrations, and the predictive gain associated with meteorological covariates was quantified through changes in *R*^2^ between the baseline and extended model configurations.

### Data quality, limitations, and environmental considerations

Although satellite-derived and reanalysis-based products provide broad spatial coverage and long temporal records, uncertainties remain when these datasets are used to represent near-surface pollutant concentrations in tropical regions. Potential sources of uncertainty include retrieval limitations, model parameterizations, and data assimilation constraints under conditions of strong convection and rapid boundary-layer evolution. These issues may affect short-term variability and the magnitude of individual peaks.

In this study, the primary objective was to diagnose temporal behavior, seasonal patterns, and meteorological modulation of SO_₂_ over a multidecadal period rather than to reproduce absolute concentrations at a specific monitoring site. Therefore, the analysis emphasizes relative changes, trend components, and covariate-driven variability, which are generally more robust than isolated daily extremes.

Despite these limitations, the combined use of CAMS/SISAM SO_₂_ fields and MERRA-2 meteorological variables provides a consistent and physically meaningful framework for investigating air pollution dynamics in regions with limited ground-based monitoring. The state-space approach further supports this objective by separating latent long-term evolution from stochastic variability and by identifying anomalous episodes that may reflect emission-driven perturbations.

## Results

### Temporal behavior of SO_2_ concentrations

Daily SO_₂_ concentrations in Campo Grande (2003–2022) exhibited a clear seasonal pattern associated with the regional tropical climate regime. While the majority of concentrations were below 10 μg m⁻^3^, peaks exceeding this level occurred, particularly during the dry season (June–September), when reduced precipitation, lower humidity, and more stable atmospheric conditions favored pollutant accumulation. The latent-level component estimated by the state-space model revealed a gradual decline in SO_₂_ concentrations over the study period, indicating an overall improvement in air quality. To support these findings, a descriptive statistics table (including mean, median, standard deviation, percentiles, minimum, and maximum values for SO_₂_ and all meteorological variables) has been added.

**Fig. 1:**
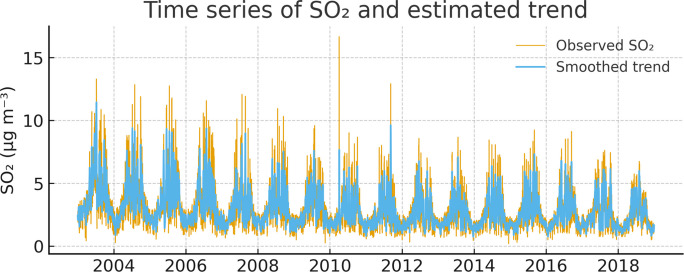
Daily SO_₂_ time series and long-term trend estimated by the state-space model

Daily SO_₂_ concentrations in Campo Grande (2003–2022) exhibited a clear seasonal pattern associated with the regional tropical climate regime. While the majority of concentrations were below 10 μg m⁻^3^ (mean = 2.53 μg m⁻^3^, median = 2.23 μg m⁻^3^, and 75th percentile = 3.25 μg m⁻^3^), peaks exceeding this level occurred, particularly during the dry season (June–September), when reduced precipitation, lower humidity, and more stable atmospheric conditions favored pollutant accumulation. The latent-level component estimated by the state space model revealed a gradual decline in SO_₂_ concentrations over the study period, indicating an overall improvement in air quality. These descriptive statistics support the findings presented in Fig. 1 and the extreme episodes identified in Table 1.

**Table 1:** Descriptive statistics of daily SO_₂_ concentrations and meteorological variables in Campo Grande, Brazil (2003–2022)

Variable	Mean	Median	Standard deviation	Minimum	Maximum	25th percentile	75th percentile
SO₂ (μg m⁻^3^)	2.53	2.23	1.76	0.42	16.67	1.35	3.25
Precipitation (mm)	3.38	0.00	7.15	0.00	73.00	0.00	4.00
Air temperature (°C)	22.66	22.65	1.96	18.15	27.78	21.00	24.30
Relative humidity (%)	68.95	70.25	16.60	28.75	97.75	55.25	83.25
Wind direction (°)	131.72	131.72	70.18	15.50	345.00	71.50	190.25
Wind speed (m s⁻^1^)	2.64	2.60	0.90	0.75	5.60	2.05	3.15

This long-term decrease is consistent with broader changes in combustion-related emissions, including reductions in sulfur content in fuels and gradual modernization of combustion technologies in Brazil. Seasonal accumulation during the dry season highlights the importance of meteorology in controlling pollutant persistence and atmospheric dilution in tropical urban environments.

Figure 1 shows the observed daily SO_₂_ time series and the long-term component estimated by the baseline state-space model.

### Influence of meteorological variables

**Fig. 2:**
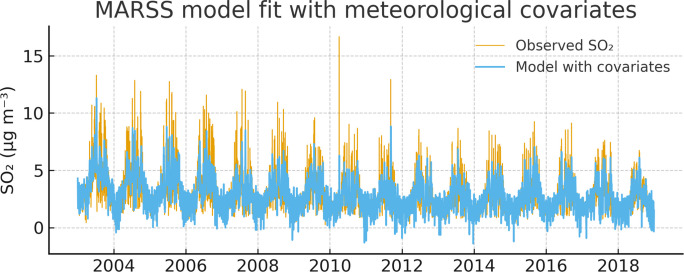
Observed SO_₂_ concentrations and MARSS model predictions with meteorological covariates

These results are consistent with established physical mechanisms governing sulfur compounds in the lower troposphere, where rainfall and humidity enhance scavenging and deposition, while wind promotes dilution and transport. Figure 2 compares observed SO₂ concentrations with predictions from the meteorology-driven MARSS model.

Table 2 presents the coefficients estimated by the MARSS model for each meteorological variable, along with the variances of the level and observation noise components. The results indicate that temperature has a positive effect on SO₂ concentrations, whereas precipitation, relative humidity, and wind speed exhibit negative coefficients, reflecting their roles in promoting dispersion and removal of the pollutant. These parameters confirm the strong influence of atmospheric conditions on the daily behavior of SO_₂_ in Campo Grande.

**Table 2:** Estimated parameters and meteorological coefficients of the MARSS model for daily SO_₂_ concentrations in Campo Grande (2003–2022)

Parameter	Description	Estimate
*σ*^2^ < sub > obs </sub >	Observation noise variance	0.75
*σ*^2^ < sub > level </sub >	State (level) noise variance	0.57
*β* < sub > precip </sub >	Precipitation coefficient	− 0.0013
*β* < sub > temp </sub >	Air temperature coefficient	+ 0.3000
*β* < sub > rh </sub >	Relative humidity coefficient	− 0.0037
*β* < sub > wind </sub >	Wind speed coefficient	− 0.7700

Table 2 presents the estimated coefficients of the meteorological covariates and the variance components of the MARSS model. Air temperature exhibited a positive association with SO_₂_ concentrations, whereas precipitation, relative humidity, and wind speed showed negative coefficients, consistent with wet removal and enhanced dispersion processes. The estimated variance terms indicate that both observation noise and state variability contributed to the overall dynamics of SO_₂_ across the study period.

These results are consistent with the physical processes governing atmospheric sulfur compounds in tropical regions, where meteorology strongly modulates daily and seasonal pollutant levels. Comparable associations have been documented in temperate and tropical cities worldwide, reinforcing the robustness of the environmental mechanisms identified (Rahman et al., 2023).

### Model performance

The performance comparison between the baseline and meteorology-driven MARSS models is summarized in Table 3. Including precipitation, air temperature, relative humidity, and wind speed increased the coefficient of determination (*R*^2^) from 0.407 to 0.573, corresponding to a relative gain of approximately 41%. Residual diagnostics indicated no significant temporal autocorrelation (Ljung–Box test at lag 30, *p* > 0.05), supporting that the temporal dependence structure was adequately captured by the model. In addition, the Kolmogorov–Smirnov and Anderson–Darling tests suggested an overall satisfactory agreement with normality assumptions.

**Table 3:** Performance comparison between MARSS models with and without meteorological covariates

Metric	Model without covariates	Model with covariates	Relative gain (%)
*R*^2^	0.407	0.573	41
Ljung–Box test (lag 30), *p*-value	> 0.05	> 0.05	–
Kolmogorov–Smirnov test, *p*-value	> 0.05	> 0.05	–
Anderson–Darling test	Acceptable	Acceptable	–

**Fig. 3:**
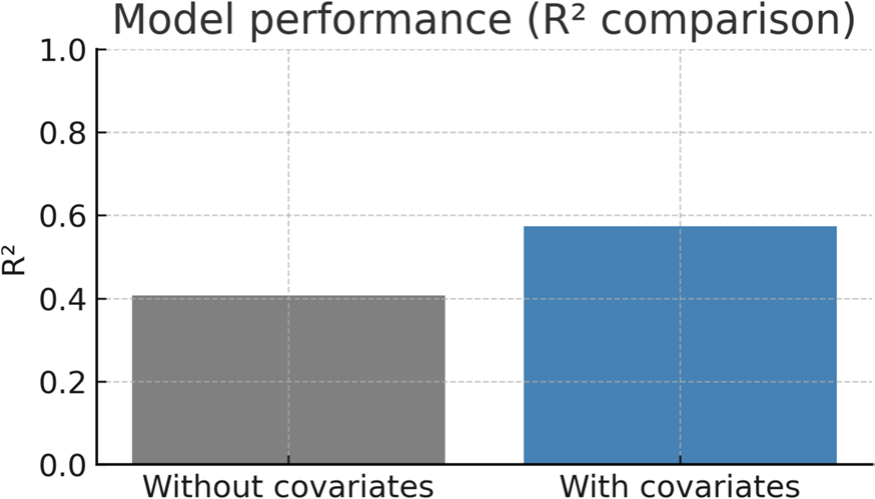
Comparison of *R*^2^ values for the baseline and extended MARSS models

Overall, these results confirm that meteorological variability explains a substantial fraction of daily SO_₂_ fluctuations in Campo Grande and that the MARSS framework provides an adequate statistical representation of pollutant dynamics in this tropical urban setting (Fig. 3).

Residual diagnostics demonstrated the following:

- no significant autocorrelation (Ljung–Box *p* > 0.05),
- adequate adherence to normality (KS and Anderson–Darling tests),
- stable variance,

confirming the statistical adequacy of the MARSS framework for SO_₂_ dynamics.

These results highlight that meteorological drivers play a dominant role in shaping short- and medium-term fluctuations in pollutant concentrations in Campo Grande.

### Identification of extreme pollution episodes

Standardized residuals from the extended MARSS model were analyzed to identify extreme SO₂ episodes not explained by meteorological conditions. Residuals exceeding the 99th percentile were interpreted as anomalous events potentially associated with short-term emission increases or processes not fully represented by the selected covariates. Most extreme episodes occurred during the dry season (July–September), when the planetary boundary layer tends to be shallower and more stable, increasing the likelihood of pollutant accumulation.

**Fig. 4:**
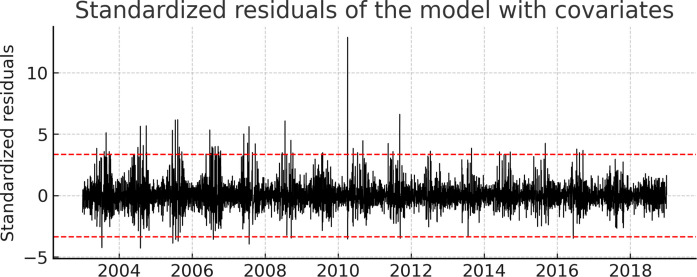
Standardized residuals of the MARSS model with meteorological covariates

These anomalies may reflect episodic emission sources such as regional biomass burning, localized combustion activities, or short-term industrial contributions. Figure 4 illustrates the standardized residual series, and the most prominent events are summarized in Table 4.

**Table 4:** Major extreme SO_₂_ pollution episodes identified from standardized residuals (> 99th percentile)

Date	Observed SO_₂_ (μg m⁻^3^)	Expected SO_₂_ (μg m⁻^3^)	Excess (μg m⁻^3^)	Standardized residual
2010-04−05	16.67	2.18	14.48	12.89
2011-09−09	12.93	5.49	7.44	6.62
2005-08−10	11.65	4.71	6.94	6.18
2005-07−18	12.75	5.83	6.92	6.16
2008-07-19	10.95	4.11	6.84	6.09

Most episodes occurred during the July–September dry season, when the regional atmospheric boundary layer becomes shallow and prone to trapping pollutants. These findings align with the seasonality of fire activity in Central Brazil and with previous studies documenting SO₂ peaks during periods of atmospheric stagnation (Chattopadhyay et al., 2010).

The identification of these anomalies emphasizes the importance of integrating emissions information—such as fire hotspots, traffic intensity, and industrial inventories—into future analyses to better distinguish meteorological influence from emission-driven events.

### Overall interpretation of SO_2_ variability

The combined analysis of state space components, meteorological covariates, and residual diagnostics provides a comprehensive characterization of SO_₂_ behavior in Campo Grande. The main findings can be summarized as follows: (i) SO_₂_ exhibited a gradual long-term decline, suggesting improved air quality over the last two decades; (ii) concentrations showed a pronounced seasonal cycle, with higher values during the dry season; (iii) meteorological variables strongly modulated daily variability, particularly through the opposing effects of temperature versus precipitation, humidity, and wind; and (iv) extreme events were primarily concentrated in the dry season and likely linked to episodic emission-driven perturbations.

Together, these results demonstrate the usefulness of MARSS models as an environmental diagnostic tool for separating long-term behavior from meteorologically driven variability and for highlighting anomalous pollution episodes in tropical urban environments.

## Discussion

The temporal behavior of SO_₂_ in Campo Grande reflects the combined influence of regional climate seasonality and emission patterns typical of tropical urban environments. The state-space trend component indicated a gradual decrease in SO_₂_ concentrations over the 2003–2022 period, suggesting an overall improvement in air quality. The magnitude of SO_₂_ concentrations observed in this study (mean ≈ 2.53 μg m⁻^3^) is consistent with values reported in urban areas based on ground-level monitoring, where typical concentrations are generally below 10–20 μg m⁻^3^ under non-polluted conditions, although higher peaks may occur during episodic emission events (Chattopadhyay et al., 2010; Rahman et al., 2023). This long-term decline is consistent with broader changes in combustion-related emissions, including progressive reductions in sulfur content in fuels and technological modernization in transportation and industrial sectors. Similar decreasing trends have been reported in other urban regions undergoing regulatory adjustments and structural changes in energy use and industrial activity (Atari et al., 2008; Wheeler et al., 2008).

Seasonality emerged as a dominant feature of SO_₂_ variability. Higher concentrations during the dry season (June–September) are consistent with the atmospheric conditions characteristic of tropical savanna climates, including reduced wet deposition, lower humidity, and a more stable boundary layer. During this period, limited convective mixing and a shallower planetary boundary layer favor pollutant accumulation near the surface. This seasonal behavior has been widely described for combustion-related pollutants in tropical and subtropical environments, where meteorology often controls short-term persistence more strongly than gradual changes in emissions (Chattopadhyay et al., 2010; Rahman et al., 2023).

Meteorological controls played a key role in shaping daily SO_₂_ fluctuations, as demonstrated by the improved performance of the MARSS model when covariates were included. Air temperature exhibited a positive association with SO_₂_, which may reflect the effect of warm and stable conditions on vertical mixing and pollutant dilution. In contrast, precipitation, relative humidity, and wind speed were negatively associated with SO_₂_ concentrations, supporting their roles in removal and dispersion processes. Precipitation is expected to reduce SO_₂_ through wet scavenging and deposition, while higher humidity can enhance aqueous-phase oxidation pathways and atmospheric cleansing. Wind speed promotes dilution and transport, reducing the residence time of SO_₂_ over the urban area. The direction and physical interpretation of these coefficients reinforce that the MARSS framework captured meaningful atmospheric mechanisms rather than purely statistical correlations, in agreement with established atmospheric chemistry principles (Seinfeld & Pandis, 2016).

The residual-based identification of extreme SO_₂_ episodes provided additional insight into emission-driven variability. Most anomalies occurred during the dry season, when meteorological conditions are less favorable for dispersion and when regional biomass burning activity typically intensifies in Central Brazil. These events likely represent short-lived emission perturbations associated with fire outbreaks, local combustion sources, or episodic industrial contributions, superimposed on the background meteorological control. The occurrence of dry-season anomalies is consistent with previous evidence that SO_₂_ peaks can emerge during stagnation conditions and in association with changes in land use and combustion activity (Chattopadhyay et al., 2010; Rahman et al., 2023).

From a methodological perspective, the MARSS approach proved useful as a diagnostic framework for separating latent long-term evolution from meteorologically driven variability and stochastic noise. Unlike static regression models, state-space structures allow the underlying level/trend to evolve over time, providing an interpretable decomposition of the series and supporting the identification of abnormal events (Harvey, 1989; Holmes & Scheuerell, 2022). This is particularly valuable in tropical regions, where pollutant dynamics are strongly influenced by seasonal rainfall regimes, rapid boundary-layer transitions, and interannual variability.

Some limitations should be acknowledged. The SO_₂_ series was derived from CAMS-based reanalysis fields accessed through SISAM/INPE rather than direct in situ monitoring. While these products provide consistent multi-decadal coverage, uncertainties may affect the representation of short-lived peaks and near-surface concentrations under rapidly evolving tropical atmospheric conditions (Christophe et al., 2019; Inness et al., 2019). However, the focus of this study was on temporal patterns, seasonal modulation, and the quantification of meteorological influence, which are generally more robust than isolated daily extremes. Despite these limitations, CAMS/SISAM datasets have been widely used for long-term assessments of reactive gases and aerosols and remain valuable for regions with limited ground-based monitoring (Eskes et al., 2015; Inness et al., 2019).

Future research could strengthen source attribution by integrating fire hotspot information, emission inventories, and traffic or industrial activity indicators. Expanding the MARSS framework to a multisite design may also help distinguish local emissions from regional transport influences, improving interpretation of SO_₂_ variability in the broader context of Central Brazil.

Overall, the results emphasize that meteorology strongly modulates SO_₂_ variability in Campo Grande, particularly during the dry season, and that state space modeling provides a practical and interpretable tool for air-quality diagnosis in regions with limited ground-based monitoring infrastructure. These findings support the development of meteorology-informed strategies for pollution mitigation and highlight the importance of targeted control measures during periods of atmospheric stability and elevated fire activity.

## Conclusion

This study investigated the temporal dynamics of daily SO_₂_ concentrations in Campo Grande, Brazil, from 2003 to 2022 using a multivariate autoregressive state space (MARSS) modeling framework and meteorological covariates from reanalysis data. The main conclusions are as follows:

(i) SO_₂_ concentrations exhibited a clear seasonal cycle, with higher values during the dry season (June–September), when atmospheric stability and reduced wet deposition favor pollutant accumulation.
(ii) A gradual decreasing long-term trend was identified over the study period, indicating an overall improvement in air quality in this tropical urban region.
(iii) Meteorological conditions explained a substantial fraction of daily SO_₂_ variability. The inclusion of precipitation, air temperature, relative humidity, and wind speed improved model performance by ~ 41% (*R*^2^ from 0.407 to 0.573), confirming the dominant role of atmospheric drivers in short-term fluctuations.
(iv) Residual analyses identified extreme SO_₂_ episodes mainly during the dry season, suggesting the influence of episodic emission events such as biomass burning and local combustion sources beyond meteorological control.

Overall, the results demonstrate that MARSS models provide an interpretable and robust framework for diagnosing pollutant variability, separating meteorological modulation from emission-driven anomalies, and supporting meteorology-informed air quality assessment in tropical regions with limited ground-based monitoring.

## Data Availability

The datasets used in this study are publicly available. Air quality data were obtained from SISAM/INPE, and meteorological variables were derived from the MERRA-2 reanalysis.

## References

[CR1] Abdul Rahim, U., & Masseran, N. (2023). State-space time series analysis on air pollution data. *Environment and Ecology Research,**11*(1), 155–164. 10.13189/eer.2023.110115

[CR2] Atari, D. O., Luginaah, I. N., Fung, K. Y., & Gorey, K. M. (2008). A land use regression model for predicting ambient sulfur dioxide concentrations in Hamilton, Ontario. *Journal of Environmental Management,**88*(3), 1053–1060. 10.1016/j.jenvman.2007.05.004

[CR3] Bascom, R. (1996). Health effects of outdoor air pollution. *American Journal of Respiratory and Critical Care Medicine,**153*(1), 3–50. 10.1164/ajrccm.153.1.8542133 8542133 10.1164/ajrccm.153.1.8542133

[CR4] Chattopadhyay, S., Chattopadhyay, G., & Chatterjee, S. (2010). Spatial variation of air pollutants and their correlation with land use patterns: A study on SO₂ and NO₂ in Kolkata, India. *Environmental Monitoring and Assessment,**160*(1–4), 297–310. 10.1007/s10661-008-0698-6

[CR5] Christophe, Y., Bennouna, Y., Schulz, M., Eskes, H., Basart, S., Benedictow, A., & Bozzo, A. (2019). Validation report of the CAMS global reanalysis of aerosols and reactive gases (2003–2018). Copernicus Atmosphere Monitoring Service. 10.24380/qs80-3t85

[CR6] Eskes, H. J., Huijnen, V., Arola, A., Benedictow, A., Blechschmidt, A. M., Botek, E., & Zhou, C. (2015). Validation of reactive gases and aerosols in the MACC global analysis and forecast system. *Geoscientific Model Development,**8*, 3523–3543. 10.5194/gmd-8-3523-2015

[CR7] Fung, K. Y., Chow, R., Lee, J., Watson, J. G., & Ho, K. F. (2005). Air pollution and daily hospitalization for cardiovascular diseases in Hong Kong. *Environmental Health Perspectives,**113*(6), 870–878. 10.1289/ehp.7672

[CR8] Gelaro, R., McCarty, W., Suárez, M. J., Todling, R., Molod, A., Takacs, L., & Zhao, B. (2017). The modern-era retrospective analysis for research and applications, version 2 (MERRA-2). *Journal of Climate,**30*(14), 5419–5454. 10.1175/JCLI-D-16-0758.1 10.1175/JCLI-D-16-0758.1 PMC6999672 32020988

[CR9] Gulliver, J., Morris, C., Lee, K., Vienneau, D., Briggs, D., & Hansell, A. (2011). Land use regression modeling to estimate long-term concentrations of air pollution: Comparison of regression models. *Atmospheric Environment,**45*(39), 6604–6613. 10.1016/j.atmosenv.2011.08.017 10.1021/es103821y PMC3076991 21446726

[CR10] Harvey, A. C. (1989). *Forecasting, structural time series models and the Kalman filter*. Cambridge University Press.

[CR11] Hoek, G., Beelen, R., de Hoogh, K., Vienneau, D., Gulliver, J., Fischer, P., & Briggs, D. (2008). A review of land-use regression models to assess spatial variation of outdoor air pollution. *Atmospheric Environment,**42*(33), 7561–7578. 10.1016/j.atmosenv.2008.05.057

[CR12] Holmes, E. E., & Scheuerell, M. D. (2022). *MARSS: *Multivariate autoregressive state-space modeling (R package, version 3.11.5). https://cran.r-project.org/package=MARSS. Accessed 15 Jan 2025.

[CR13] Inness, A., Ades, M., Agustí-Panareda, A., Barré, J., Benedictow, A., Blechschmidt, A. M., & Zhang, C. (2019). The CAMS reanalysis of atmospheric composition. *Atmospheric Chemistry and Physics,**19*(6), 3515–3556. 10.5194/acp-19-3515-2019

[CR14] Instituto Nacional de Pesquisas Espaciais. (2024). SISAM—Sistema de Informação de Saúde Ambiental. https://dataserver-coids.inpe.br/queimadas/queimadas/sisam/. Accessed 12 Jan 2025.

[CR15] Kanaroglou, P. S., Adams, M. D., De Luca, P. F., Corr, D., & Sohel, N. (2013). Estimation of sulfur dioxide air pollution concentrations with a spatial autoregressive model. *Atmospheric Environment,**79*, 421–427. 10.1016/j.atmosenv.2013.07.014

[CR16] Medley, G. F., Anderson, R. M., Lam, T. H., Wong, C. M., & Hedley, A. J. (2002). Short-term effects of air pollution on daily mortality in Hong Kong. *Environmental Research,**90*(3), 260–268. 10.1006/enrs.2002.4416

[CR17] Mercer, L. D., Szpiro, A. A., Sheppard, L., Lindström, J., Adar, S. D., & Kaufman, J. D. (2011). Comparing universal kriging and land-use regression for estimating long-term particulate air pollution concentrations. *Atmospheric Environment,**45*(26), 4474–4484. 10.1016/j.atmosenv.2011.05.043 10.1016/j.atmosenv.2011.05.043 PMC3146303 21808599

[CR18] Park, E. J., Lee, K., Kim, H., Lee, S., & Park, J. (2010). Short-term effects of air pollution on mortality: Results from multi-city time-series studies in Korea. *Environmental Research,**110*(3), 257–262. 10.1016/j.envres.2009.12.003

[CR19] Rahman, M. M., Karim, M. N., Khan, M. S. H., Rahman, M. A., & Kabir, M. H. (2023). Comprehensive evaluation of spatial distribution and temporal variation of key air pollutants, including sulfur dioxide (SO_2_), using remote sensing and ground-based observations. *Remote Sensing,**15*(20), Article 5069. 10.3390/rs15205069

[CR20] Seinfeld, J. H., & Pandis, S. N. (2016). *Atmospheric chemistry and physics: From air pollution to climate change* (3rd ed.). John Wiley & Sons.

[CR21] Wheeler, A. J., Smith-ron, M., Xu, X., Gilbert, N. L., Brook, J. R., & Brook, R. D. (2008). Intra-community variability of air pollution in Canadian urban areas. *Atmospheric Environment,**42*(39), 8885–8895. 10.1016/j.atmosenv.2008.06.045

[CR22] World Health Organization. (2021). *WHO global air quality guidelines: Particulate matter (PM₂.₅ and PM₁₀), ozone, nitrogen dioxide, sulfur dioxide and carbon monoxide*. World Health Organization. 34662007

